# Determination of optimal NH_4_^+^/K ^+^ concentration and corresponding ratio critical for growth of tobacco seedlings in a hydroponic system

**DOI:** 10.3389/fpls.2023.1152817

**Published:** 2023-07-11

**Authors:** Chuanzong Li, Oluwaseun Olayemi Aluko, Sujuan Shi, Zhijie Mo, Tongjia Nong, Chuhan Shi, Zhihao Li, Qian Wang, Haobao Liu

**Affiliations:** ^1^ Tobacco Research Institute, Chinese Academy of Agricultural Sciences, Qingdao, China; ^2^ State Key Laboratory of Plant Stress Biology, School of Life Sciences, Henan University, Kaifeng, China; ^3^ Technology Center, Shanghai Tobacco Company, Ltd, Beijing, China; ^4^ Yichang City Company, Hubei Tobacco Company, Yichang, China

**Keywords:** NH_4_^+^ -K^+^ concentrations, NH_4_^+^: K^+^ ratios, tobacco seedling growth, NH_4_^+^ toxicity, tobacco plant organs (leaf, stem, and root)

## Abstract

Inherently, ammonium (NH_4_^+^) is critical for plant growth; however, its toxicity suppresses potassium (K^+^) uptake and vice-versa. Hence, attaining a nutritional balance between these two ions (NH_4_^+^ and K^+^) becomes imperative for the growth of tobacco seedlings. Therefore, we conducted a 15-day experimental study on tobacco seedlings exposed to different concentrations (47 treatments) of NH_4_^+^/K^+^ at different corresponding 12 ratios simultaneously in a hydroponic system. Our study aimed at establishing the optimal NH_4_^+^-K^+^ concentration and the corresponding ratio required for optimal growth of different tobacco plant organs during the seedling stage. The controls were the baseline for comparison in this study. Plants with low or excessive NH_4_^+^-K^+^ concentration had leaf chlorosis or dark greenish colouration, stunted whole plant part biomass, and thin roots. We found that adequate K^+^ supply is a pragmatic way to mitigate NH_4_^+^-induced toxicity in tobacco plants. The optimal growth for tobacco leaf and root was attained at NH_4_^+^-K^+^ concentrations 2-2 mM (ratio 1:1), whereas stem growth was optimal at NH_4_^+^-K^+^ 1-2 mM (1:2). The study provided an insight into the right combination of NH_4_^+^/K^+^ that could mitigate or prevent NH_4_^+^ or K^+^ stress in the tobacco seedlings.

## Introduction

Ammonium (NH_4_^+^) is a predominant form of nitrogen (N) that supports plant growth, especially when furnished with an adequate amount of nitrate (NO_3_^-^) (Dubey et al., 2021; Vu et al., 2021; Zhu et al., 2021; Ninkuu et al., 2023). Compared to sole NO_3_^-^ or NH_4_^+^, crop growth and yield peaks when NH_4_^+^ and NO_3_^-^ are appropriately combined in growth medium (Liang et al., 2022; Saloner and Bernstein, 2022; Xiao et al., 2023). However, compelling evidence has shown that excessive or sole application of NH_4_^+^ could negate plant growth and yield potentials, causing leaf chlorosis, stunted growth, and other major crop physiological disorder (Britto and Kronzucker, 2002; Guo et al., 2019; Aluko et al., 2021; Katz et al., 2022). Over time, the mechanisms underlying NH_4_^+^ toxicity in plants have been debatable, with much speculations accrued to the displacement of cytosolic cations, including K^+^ (Walch-Liu et al., 2000; Guo et al., 2019).

Given that, the interactive effects of K^+^ and NH_4_^+^ on plant growth and development became a focus of research (Witold et al., 2017; Shi et al., 2020a; Nawarathna et al., 2021; Aluko et al., 2022; Xiao et al., 2023). Excessive NH_4_^+^ was found to limit growth and yield by reducing K^+^ uptake and influx in wheat (Kong et al., 2014), *Arabidopsis* (Walch-Liu et al., 2000), rice (Szczerba et al., 2008) and tobacco (Lu et al., 2005). Research has shown the potency of potassium in alleviating NH_4_^+^ toxicity symptoms in these crops. (Chen et al., 2021; Fang et al., 2021). Recently, Guo et al. (2019) reported a marked increase in the growth, nutrient uptake, and yield (improved panicle biomass production) when K^+^ concentration was supplied to high NH_4_^+^- induced wheat, demonstrating the positive impact of K^+^ in offsetting NH_4_^+^ stress in plants. Moreover, excessive application of K^+^ could hinder the uptake of NH_4_^+^, resulting to a competition in the uptake of both essential ions (K^+^ and NH_4_^+^) (Balkos et al., 2010). Disruption in the uptake of either of these two cations limit plant growth potentials. Despite the promising results of K^+^ ameliorative effects on NH_4_^+^, it is yet unknown the appropriate amount of NH_4_^+^ and K^+^ required for optimal yield, knowledge of which is important given the increasing NH_4_^+^ toxicity symptoms in plants. Hence, an in-depth understanding of a nutritional balance between NH_4_^+^ and K^+^ becomes expedient.

Tobacco plants require an adequate supply of K^+^ for improved growth and quality (Borges et al., 2012). Similarly, supply of NH_4_^+^ further promote growth, but becomes toxic if excessive. However, the issue of nutritional imbalances between K^+^ and NH_4_^+^ poses a great challenge to adopting a hydroponic system for tobacco cultivation. Hence, it becomes imperative to understand the appropriate NH_4_^+^/K^+^ concentration and the corresponding ratio required for the growth of tobacco. Research-based information on the basic NH_4_^+^/K^+^ concentration and corresponding ratio required for optimal growth of tobacco under a hydroponic system is still lacking. Moreover, little is known about the influence of increasing NH_4_^+^ and K^+^ supplies on the physiological growth of tobacco plants. Hence, an in-depth understanding of tobacco’s optimum NH_4_^+^- K^+^ concentration range is required to furnish hydroponic farmers with reliable information on the best NH_4_^+^/K^+^ combination to attain optimum growth output. This study aimed to investigate the effects of different levels of NH_4_^+^- K^+^ concentration on the growth of tobacco seedlings, and also critically evaluate and determine the optimal NH_4_^+^ - K^+^ concentration, and corresponding ratio that is required for the growth of different parts of tobacco seedlings.

## Materials and methods

### Plant materials and growth conditions

The experiment was conducted in a controlled plant growth culture room at Tobacco Research Institute, Chinese Academy of Agricultural Sciences (TR1,CAAS), Qingdao, China. All methods used in this study were performed in accordance with the relevant guidelines and regulations. The seed of *Nicotiana tabacum* used in the present study was Zhongyan 100 (ZY100). ZY100 developed in TR1CAAS passed the variety approval in 2002. Tobacco seeds were initially sown in a potting soil mixture (soil/perlite, 3:1 v/v) under controlled conditions (continuous light at 24°C). At the three-leaf stage, uniformly grown seedlings were transferred into hydroponic pots (48 cm x 22.5 cm x 3.5 cm) with 2 liters of nutrient solution (one-fifth-strength Hoagland solution, 1/5 HS) for 6 days. The 1/5 HS, which was supplemented with 1mM K^+^ (K_2_SO_4_ is the K^+^ source) had the following composition in mM: 0.35 MgSO_4_, 0.2 NaH_2_PO_4_, 0.0125 H_3_BO_3_, 0.001 MnSO_4_, 0.0005 CuSO_4_, 0.001 ZnSO_4_, 0.0001 Na_2_MoO_4_, 0.01 Fe-EDTA, 1.4 Ca (NO_3_)_2_, 0.15 CaCl_2_. NH_4_^+^ was sourced from (NH_4_)_2_SO_4_.

### NH_4_^+^ and K^+^ treatments

After six days, tobacco seedlings with the same growth potential were transferred into another hydroponic container (26 cm x 17.5 cm x 8 cm) of 1/5 HS. NH_4_^+^ and K^+^ were supplied depending on the designated NH_4_^+^- K^+^ concentration in each treatment group. Seedlings were held in place by the conventional tip and grown at 24°C. The experiment was sectioned into two NH_4_^+^/K^+^ categories. The first category was fractioned into increasing NH_4_^+^ at constant K^+^, comprising 7 ratio, while the other, constant NH_4_^+/^increasing K^+^ had 5 ratios, giving 12 ratios in total. The 12 NH_4_^+^: K^+^ ratios comprise 45 different NH_4_^+^-K^+^ concentrations, and two additional controls (positive and negative control), bringing the total to 47 different NH_4_^+^-K^+^ concentrations (for composition; refer to **Table 1**). While the nutrient solution of the negative control was neither supplied with K^+^ nor NH_4_^+^, the medium with the positive control was supplemented with 1 mM K^+^ but no NH_4_^+^ supply. Tobacco seedlings were exposed to varying concentrations (47 treatments) of NH_4_^+^/K^+^ at different corresponding 12 ratios concurrently in a hydroponic system for 15 days. The nutrient solution was renewed every two days to ensure a steady nutritional state for tobacco seedlings. The solution pH was maintained between 5.6 and 6.0. The placements of the hydroponic pots were interchanged to avoid edge effects.

**Table 1:** NH_4_^+^/K^+^ concentrations with their corresponding ratios.

Ratios NH_4_^+^: K^+^	NH_4_^+^-K^+^ treatments (mM)
1:1	0.1-0.1、0.5-0.5、1-1、2-2、5-5、10-10
2:1	0.2-0.1、1-0.5、2-1、10-5、20-10
5:1	0.5-0.1、1-0.2、5-1、10-2、50-10
10:1	1-0.1、5-0.5、10-1、20-2
20:1	2-0.1、10-0.5、20-1
50:1	5-0.1、10-0.2、50-1
100:1	10-0.1、20-0.2、50-0.5
1:2	0.1-0.2、0.5-1、1-2、5-10
1:5	0.1-0.5、0.2-1、1-5、2-10
1:10	0.1-1、0.2-2、0.5-5、1-10
1:20	0.1-2、0.5-10
1:50	0.1-5、0.2-10
Positive control Negative control	0-0.5 0-0

NH_4_^+^-K^+^; millimolar: (mM)).

We had a pre-experimental trial, with the plants exceeding 15 days of NH_4_^+^- K^+^ treatments. when plants exceeded the 15th day, tobacco wilting and overall death were observed due to the excessive supply of NH_4_^+^ or K^+^ to some of the treated samples. As such, some samples were not available for phenotype analysis. Therefore, we considered sampling the tobacco seedlings at 15 days after treatment for thorough evaluation of the physiological parameters.

. After 15 days of treatment, tobacco seedlings were harvested for leaf, stem, and root growth, NH_4_^+^ and K^+^ content, and root activity analysis.

### Plant biomass, leaf and root surface area, stem and root length

At harvest, uniformly grown seedlings from each treatment were fractioned into; (i) leaves, (ii) stems, and (iii) roots. Photos of different plant parts were taken with a camera. Subsequently, leaf and root surface area were determined using the ImageJ software (https://imagej.en.softonic.com/ ). Primary root length and stem length were measured with a scaling ruler. Plant root was carefully rinsed once with 10 mM CaSO_4_ and twice in double-distilled water (Szczerba et al., 2008), and then fresh weights of leaves, stems, and roots of plants were measured. The dry weights of the measured samples were taken after oven-drying at 110°C for 30 min and then 80 °C to a constant weight. The dry samples were crushed into fine powders with the mortar and pestle for K^+^ concentrations determination.

### K^+^ and NH_4_^+^ content measurements

To measure K^+^ content, approximately 0.01 g of ground leaves, stems, and roots samples were weighed and digested in 8 ml 0.5 M HCl. The suspension was homogenized at 25°C, 100-150 rpm for 1hr and filtered into a new centrifuge tube. The aliquot of the filtrate was used for K^+^ determination by flame photometry (6400A) (Shi et al., 2020a). The reading obtained was used to calculate K^+^ concentrations in plant tissue as follows:

K^+^ (mmol g^-1^ DW) = ((A/M) * V *Dilution multiples*0.001)/m

where A = calculated concentration according to the readings on the standard curve (µg·ml^-1^)

M = relative molecular mass of K^+^

V = reading volume (ml)

m = dry weight (g)

For NH_4_^+^ content measurement, the freshly harvested plant was separated into different plant parts (leaves, stems, and roots). The root was washed with 10mM CaSO_4_ to eliminate any extracellular NH_4_^+^ (Szczerba et al., 2008). Fresh plant tissues of ≤ 0.5 g were homogenized under liquid nitrogen, and 6 ml of 10 mM formic acid was added to extract NH_4_^+^. The suspension was allowed to sit for 5 minutes and then centrifuged at 4°C and 12000 rpm for 10 minutes. The supernatant was centrifuged repeatedly 3 times. The supernatant obtained from the last centrifugation step was diluted with 2.5 ml o-phthalaldehyde (OPA) solution as previously described (Shi et al., 2020a). The absorbance of the sample was measured at 410 nm using a spectrophotometer (UV-7502PC, AOE Instruments). The reading obtained was used to calculate NH_4_^+^ concentrations in plant tissue as follows:

NH_4_^+^ (µmol g^-1^ FW) = ((A/M) * V * Dilution multiples)/m
where A = calculated concentration according to the readings on the standard curve (µg ml^-1^)
M = relative molecular mass of NH_4_^+^;
V = reading volume (ml)
m = fresh weight (g).

### Chlorophyll content measurement

After 15 days of NH_4_^+^/K^+^ treatment, chlorophyll content was measured according to the previous method (Dong et al., 2015). The fourth leaf of each treatment was weighed (0.2 g) and incubated in 95% ethyl alcohol until the leaf strands became completely pale (approximately 48 hours). The absorbance of the extract was measured at 665 nm and 649 nm using a spectrophotometer.

### Root activity assays

Root activity was measured as described previously (Liu et al., 2008) using triphenyl tetrazolium chloride (TTC) method. Approximately 0.5 grams of freshly weighed root was fully immersed into 5 ml 0.4% TTC and phosphate buffer (adjusted to pH 7.0) and incubated at 37 °C for 3 hours to accelerate the reduction of TTC to triphenyl formazan (TTF). The resulting chemical reaction was halted by adding 2 ml of 1 mol/L sulphuric acid into each tube. Subsequently, the roots were moved out of the tubes, gently patted with tissue paper, and then crushed with 3-4 ml ethyl acetate. The liquid portion was removed into a new tube and made up to 10 ml with ethyl acetate. The absorbance was measured at 485 nm wavelength using spectrometer UV-7502PC, AOE Instruments). The OD values were expressed as mg TTF/(g·h).

### Statistical analysis

Data was analyzed using the IBM SPSS Statistics 23 software. Variations among treatments were examined by one-way ANOVA, and means were compared using Duncan’s multiple range tests at P<0.05. All graphs were drawn using GraphPad Prism 6.0. The correlation analysis was performed using pearson correlation in R studio. ns: no significance difference; *p < 0.05; **p < 0.01; ***p < 0.001.

## Results

### Leaf growth of tobacco seedlings as affected by varying NH_4_^+^-K^+^ concentrations and ratios

**Figure 1:**
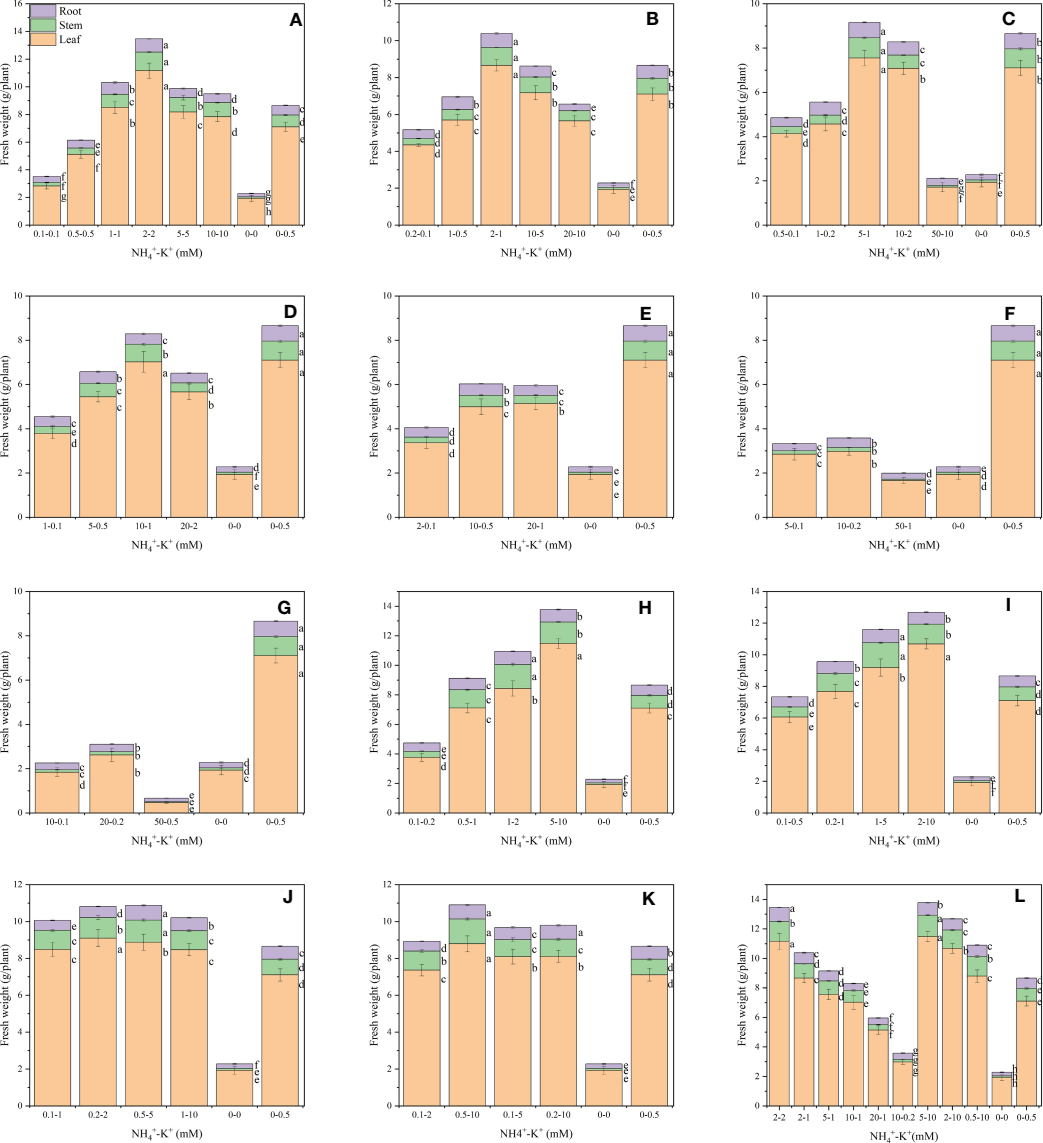
Fresh leaf weight (FLW), fresh stem weight (FSW), and fresh root weight (FRW) of tobacco plants as affected by different NH_4_^+^: K^+^ ratios. Graphs **(A–G)** represent NH_4_^+^-K^+^ concentrations at increasing NH_4_^+^/constant K^+^ (at 1) ratios. **(A)** NH_4_^+^: K^+^ ratio 1:1, **(B)** 2:1, **(C)** 5:1, **(D)** 10:1, **(E)** 20:1, **(F)** 50:1, and **(G)** 100:1. Graphs **(H-K)** indicate NH_4_^+^-K^+^ concentrations at constant NH_4_^+^ (at 1)/increasing K^+^ ratio. **(H)** NH_4_^+^: K^+^ ratio 1:2, **(I)** 1:5, **(J)** 1:10, **(K)** 1:20 (0.1-2 mM and 0.5-10 mM) and 1:50 (0.1-5 mM and 0.2-10 mM), **(L)** comparison of NH_4_^+^-K^+^ concentration within each ratio. All the NH_4_^+^-K^+^ concentration within each ratio were compared with the positive (without NH_4_^+^, but with K^+^).and negative control (without NH_4_^+^ and K^+^). Fresh weights were determined 15 days after treatment. Letters represent the mean values ± SD (n=15 biological replicates). The bars without letters have extremely low mean values.

The observed results showed the influence of NH_4_^+^-K^+^ concentrations on fresh leaf weight (**Figure 1**). There were variations within treatments in all NH_4_^+^: K^+^ ratios. Within each of the increasing NH_4_^+^: constant K^+^ ratios, fresh leaf weight increases with increasing NH_4_^+^ -increasing K^+^ concentration until a point was reached where further increase led to a gradual reduction. NH_4_^+^-K^+^ millimolar concentration 2-2 mM (within1:1), 2-1 mM (within 2:1) and 5-1 mM (within 5:1) had the highest weight, with 57.7%, 21.7% and 6.5% increase in fresh leaf weight, respectively, compared with the positive control (0.5 mM K^+^, without NH_4_^+^). NH_4_^+^-K^+^ concentrations 0.1-0.1 mM, 0.2-0.1 mM and 50-10 mM had the lowest leaf weight following the same ratio pattern above. The fresh leaf weight of 0.5-0.5 mM at ratio 1:1 was also significantly lower than the positive control (0 mM NH_4_^+^- 0.5 mM K^+^) but higher than the negative control (no NH_4_^+^- K^+^). The leaf weight of the positive control was substantially higher than all the NH_4_^+^-K^+^ concentrations at ratios 2:1 and 5:1, with the exemption of 2-1 mM (2:1) and 5-1 mM (5:1). Compared with other concentrations within ratios 10:1, 20:1, 50:1, and 100:1, the positive control (0.5 mM K^+^, without NH_4_^+^) had the highest leaf weight. In fact, 50-1 mM (50:1) and 50-0.5 mM (100:1) were either insignificant or 75.2% lower than the negative control (without NH_4_^+^ and K^+^). When NH_4_^+^ was kept constant (at 1) at an increasing K^+^ ratio, the leaf fresh weight of NH_4_^+^: K^+^ ratios 1:2, 1:5, and 1:20 increased with increasing NH_4_^+^/K^+^ millimolar concentration, albeit concentrations at ratios 1:10 and 1:50 were exceptions to this. Following the same ratio pattern, the leaf weight of NH_4_^+^-K^+^ concentrations was substantially higher than both controls. Yet, the leaf weight of lower treatments such as 0.1-0.2 mM (1:2) and 0.1-0.5 mM (1:5) was drastically reduced by 47.1% and 15.2% compared with the positive control. With the exemption of 5-10 mM, all other treated concentrations under increasing K^+^ ratios had considerably lower leaf weight values relative to 2-2 mM (1:1). NH_4_^+^-K^+^ 5-10 mM (within a 1:2 ratio) had the highest leaf weight; however, when compared with 2-2 mM (within ratio 1:1; which was the highest under increasing NH_4_^+^/constant K (1)), 5-10 mM was higher, although at a small difference of 2.9%.

**Figure 2:**
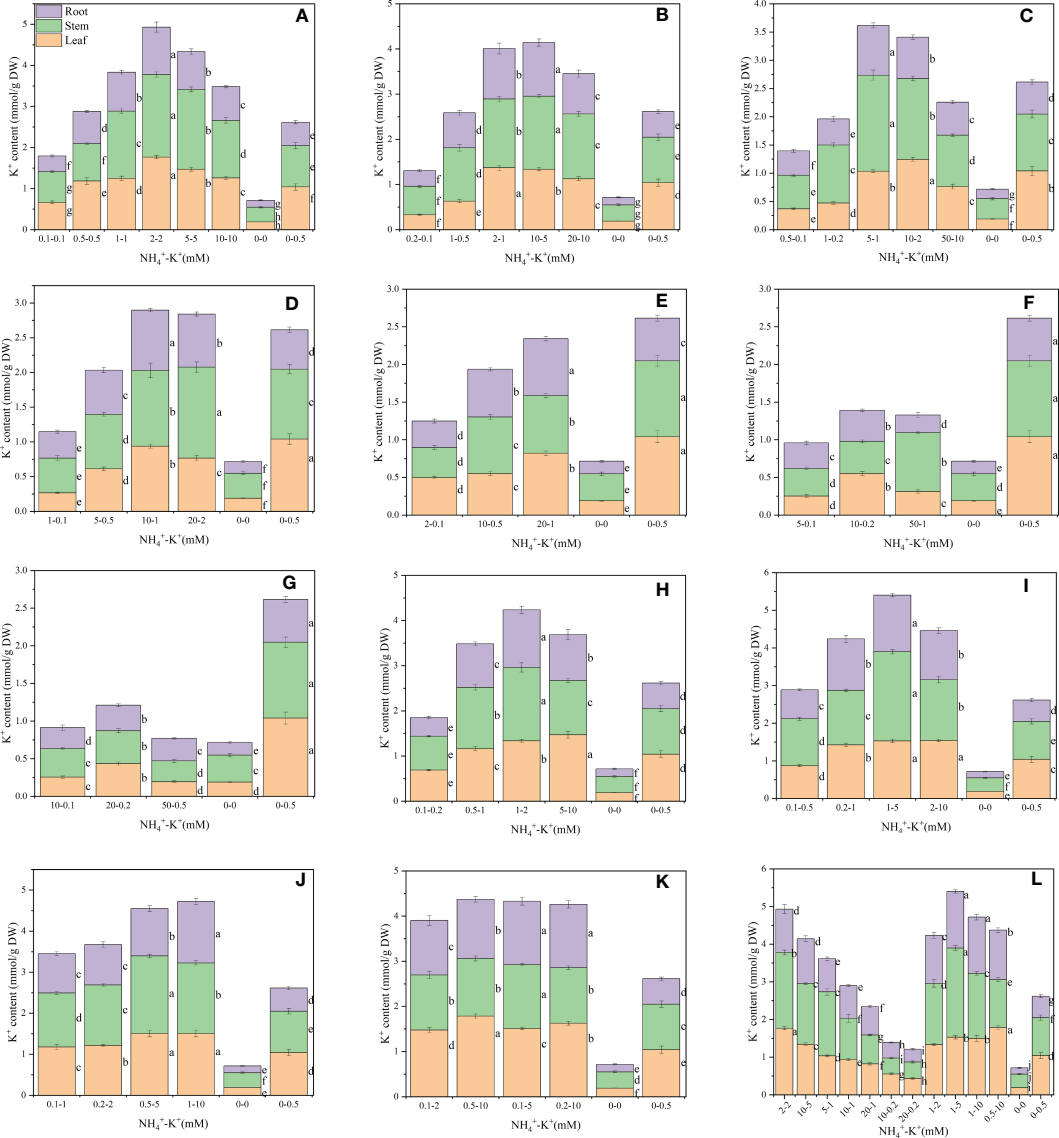
Potassium (K^+^) contents in the leaf (LKC), stem (SKC) and root (RKC) of tobacco subjected to different NH_4_^+^: K^+^ ratios. Graphs **(A-G)** represent NH_4_^+^- K^+^ concentrations at increasing NH_4_^+^/constant K^+^ (at 1) ratios. **(A)** NH_4_^+^: K^+^ ratio 1:1, **(B)** 2:1, **(C)** 5:1, **(D)** 10:1, **(E)** 20:1, **(F)** 50:1, and **(G)** 100:1. Graphs **(H-K)** connote NH_4_^+^- K^+^ concentrations at constant NH_4_^+^ (at 1)/increasing K^+^ ratio. **(H)** NH_4_^+^: K^+^ ratio 1:2, **(I)** 1:5, **(J)** 1:10, **(K)** 1:20 (0.1-2 mM and 0.5-10 mM) and 1:50 (0.1-5 mM and 0.2-10 mM) **(L)** comparison of NH_4_^+^-K^+^ concentration within each ratio. All the NH_4_^+^- K^+^ concentration within each ratio were compared with the positive (without NH_4_^+^, but with K^+^).and negative control (without NH_4_^+^ and K^+^). K^+^ content was measured 15 days after treatment. Letters represent the mean values + SD (n= 12 biological replicates).

NH_4_^+^-K^+^ concentrations at different ratios also significantly affected the leaf K^+^ content (**Figure 2**). Within each of the increasing NH_4_^+^: constant K^+^ ratios, leaf K^+^ content increases with increasing NH_4_^+^- increasing K^+^ concentration until a point is reached where further increase results in a gradual reduction. The leaf K^+^ content of 2-2 mM, 2-1 mM,10-2 mM at ratios 1:1, 2:1 and 5:1, respectively, were increased by 69.7%, 31.7% and 19.6% respectively, when compared with the positive control. Also, the leaf K^+^ contents were reduced at lower NH_4_^+^-K^+^ concentration 0.1-0.1 mM (1:1), 0.2-0.1 mM and 1-0.5 mM (2:1) relative to the positive control. At ratios 5:1, the leaf K^+^ content of the positive control was significantly higher than all other treated concentrations, except 5-1 mM. All other concentrations at ratios 10:1, 20:1, 50:1, and 100:1 exhibit reduced K^+^ contents compared with the positive control. When K^+^ was increased at constant NH_4_^+^ (1), K^+^ content increased with increasing NH_4_^+^-K^+^ millimolar concentration, irrespective of the controls (positive and negative). At such increasing K^+^ ratios, only the leaf K^+^ content of the lower concentration, 0.1-0.2 (1:2) and 0.1-0.5 (1:5), were reduced relative to the positive control; other NH_4_^+^-K^+^ treatments within these ratios were higher. Comparing the highest at both ends (constant NH_4_^+^/increasing K^+^ and increasing NH_4_^+^/constant K^+^ ratios), 0.5-10 mM (1:20) had the highest, and the leaf K^+^ content was approximately 1% higher than 2-2 mM (1:1; under increasing NH_4_^+^ and constant K^+^).

**Figure 3:**
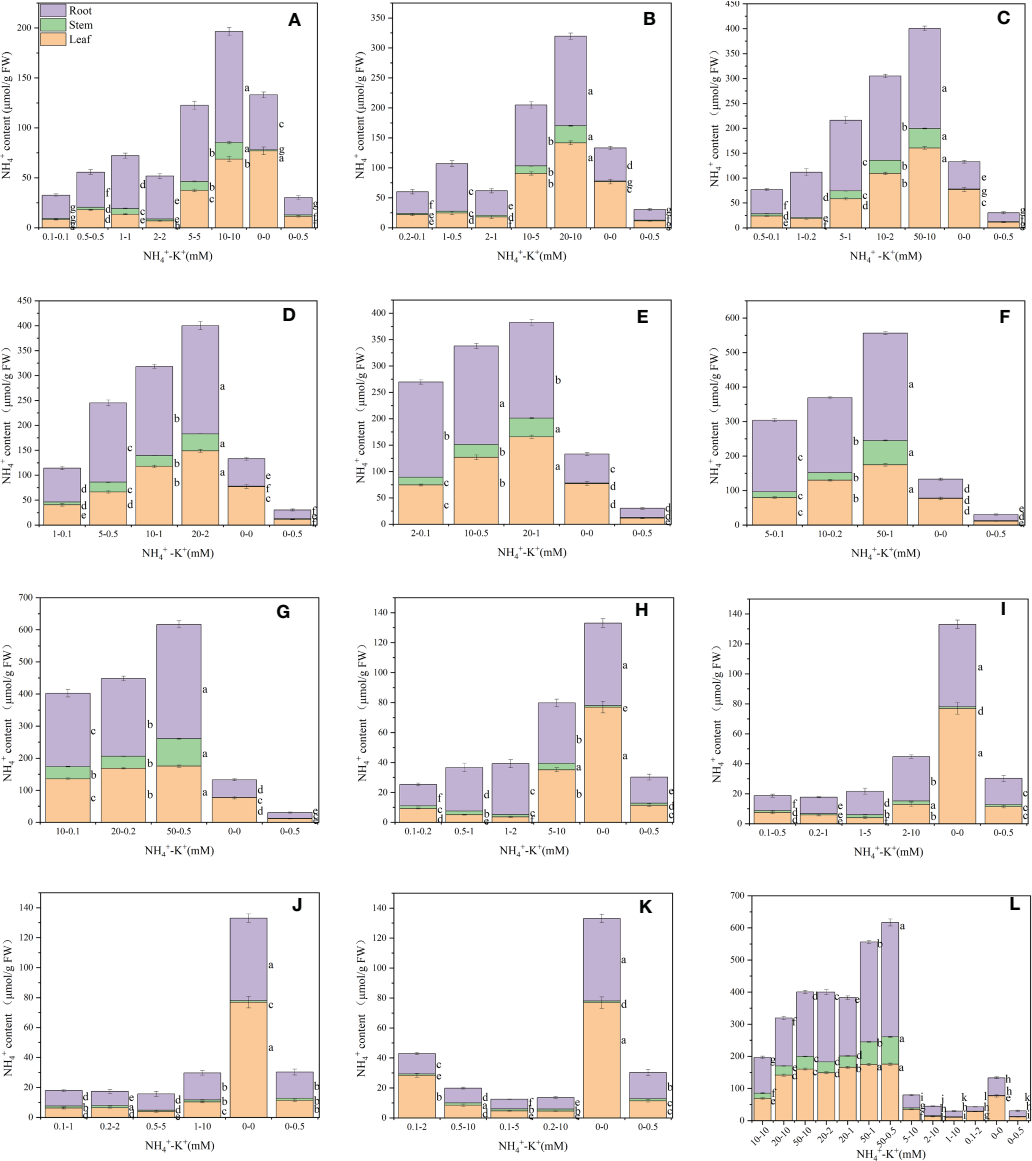
Ammonium (NH_4_^+^) contents in the leaf, stem and root of tobacco subjected to different NH_4_^+^: K^+^ ratios. Graphs **(A-G)** represent increasing NH_4_^+^ at constant K^+^ (at 1) ratios. **(A)** NH_4_^+^: K^+^ ratio 1:1, **(B)** 2:1, **(C)** 5:1, **(D)** 10:1, **(E)** 20:1, **(F)** 50:1, and **(G)** 100:1. Graphs **(H–K)** connote constant NH_4_^+^ (at 1) at increasing K^+^ ratio. **(H)** NH_4_^+^: K^+^ ratio 1:2, **(I)** 1:5, **(J)** 1:10, **(K)** 1:20 (0.1-2 mM and 0.5-10 mM) and 1:50 (0.1-5 mM and 0.2-10 mM) **(L)** comparison of NH_4_^+^-K^+^ concentration within each ratio. All the NH_4_^+^- K^+^ concentration within each ratio were compared with the positive (without NH_4_^+^, but with K^+^).and negative control (without NH_4_^+^ and K^+^). NH_4_^+^ content was measured 15 days after treatment. Letters represent the mean values + SD (n= 9 biological replicates). The bars without letters have extremely low mean values.

Leaf NH_4_^+^ content was determined to evaluate the optimal NH_4_^+^-K^+^ concentration required for the growth of tobacco seedlings. There were variations among concentrations at different ratios. At ratios 1:1, 2:1 and 5:1, leaf NH_4_^+^ content increases with increasing NH_4_^+^-K^+^ millimolar concentration until a point was reached where a notable decrease in NH_4_^+^ content was observed (NH_4_^+^-K^+^ 2-2Mm (ratio 1:1); 2-1mM (2:1); 1-0.2mM (5:1). A further increase in NH_4_^+^-K^+^ millimolar concentration resulted in a surge in leaf NH_4_^+^ content. However, at ratios 10:1,20:1, 50:1 and 100:1, a steady rise in leaf NH_4_^+^ content was observed with increasing NH_4_^+^-K^+^ concentration. The concentrations within these ratios were significantly lower than the positive control (0-0.5 mM) (**Figure 3**). Compared to the treated concentration at a constant NH_4_^+^/increasing K^+^ ratio, treatments without NH_4_^+^ and K^+^ (negative control) had the highest leaf NH_4_^+^ content. In all, the leaf NH_4_^+^ content was highest at NH_4_^+^-K^+^ concentration 50-1 mM (72.7 µmol/g FW).

**Figure 4:**
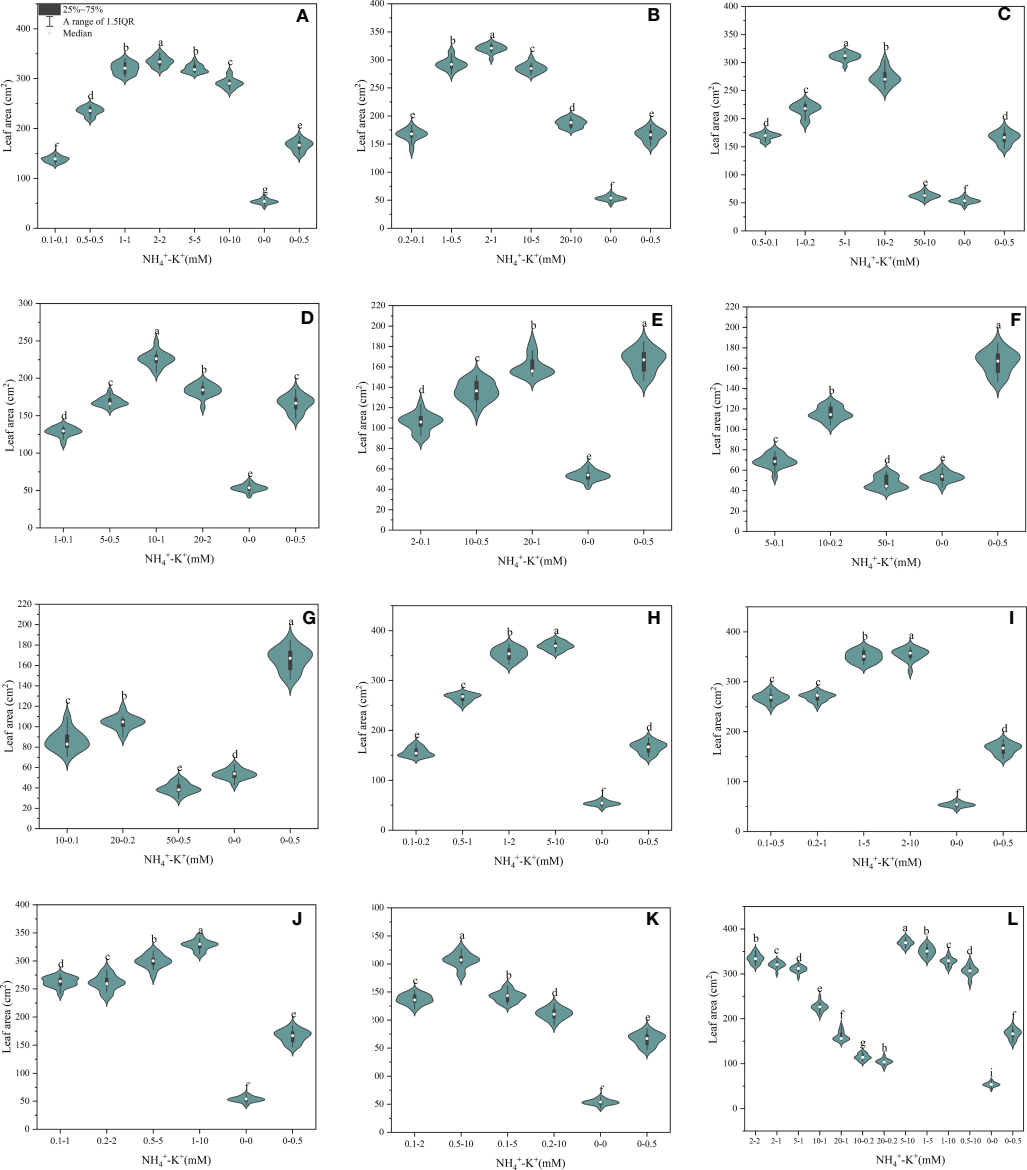
Leaf area of tobacco as affected by different NH_4_^+^: K^+^ ratios. Graphs **(A–G)** represent increasing NH_4_^+^ at constant K^+^ (at 1) ratios. **(A)** NH_4_^+^/K^+^ ratio 1:1, **(B)** 2:1, **(C)** 5:1, **(D)** 10:1, **(E)** 20:1, **(F)** 50:1, and **(G)** 100:1. Graphs **(H–K)** connote constant NH_4_^+^ (at 1) at increasing K^+^ ratio. **(H)** NH_4_^+^/K^+^ ratio 1:2, **(I)** 1:5, **(J)** 1:10, **(K)** 1:20 (0.1-2 mM and 0.5-10 mM) and 1:50 (0.1-5 mM and 0.2-10 mM) **(L)** comparison of NH_4_^+^-K^+^ concentration within each ratio. All the NH_4_^+^/K^+^ concentration within each ratio were compared with the positive (without NH_4_^+^, but with K^+^) and negative control (without NH_4_^+^ and K^+^). The above growth parameters were measured 15 days after treatment. Significant means were separated using standard deviation ± SD (n= 12 biological replicates).

There were significant differences in the leaf area of tobacco seedlings exposed to varying NH_4_^+^-K^+^ concentrations, as presented in **Figure 4**. Within each of the increasing NH_4_^+^/constant K^+^ (1) ratios (irrespective of both controls), leaf area increases with increasing NH_4_^+^-K+ millimolar concentration until a point is reached where further increase led to a gradual reduction in leaf area. Following the same ratio pattern, leaf area peaks at concentration 2-2 mM (1:1), followed by 2-1 mM (2:1), 5-1 mM (5:1), and 10-1 mM (10:1), but was significantly reduced at ratios 20:1, 50:1 and 100:1 compared with the positive control (**Figure 4**). However, at constant NH_4_^+^/increasing K^+^, a direct relationship was observed between the treated concentrations and leaf area; leaf area increases as NH_4_^+^/K^+^ concentration increases. With the exemption of 0.1-0.2 mM (1:2), the leaf area of all other treated concentrations was significantly increased relative to the positive control. In all, leaf area of concentrations 5-10 mM (1:2) and 2-10 mM (1:5) were substantially increased by 10.5% and 6.1% relative to 2-2 mM (1:1; with the highest leaf area under increasing NH_4_^+^/constant K^+^) while the negative control had the lowest leaf area.

**Figure 5:**
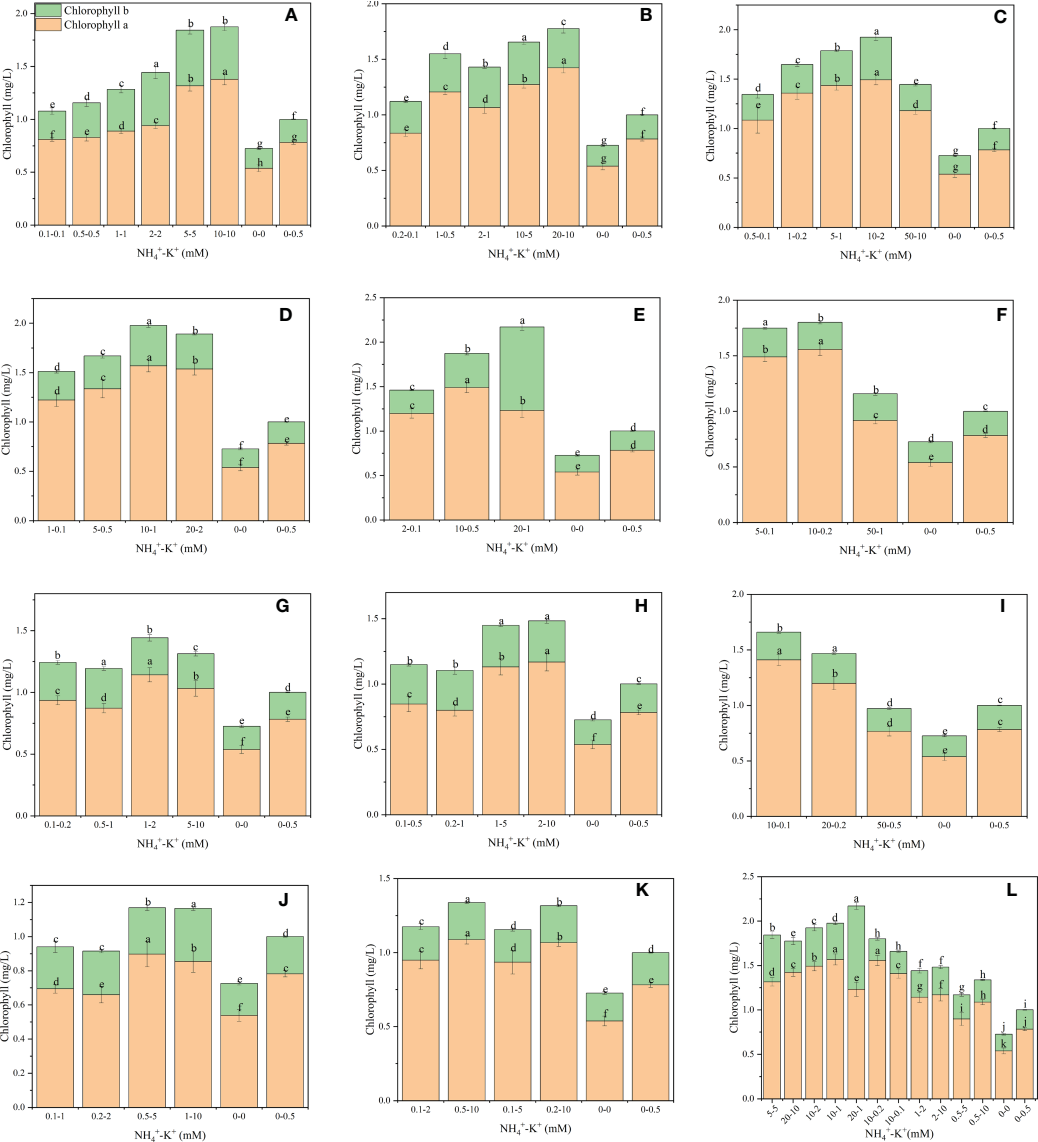
Chlorophyll contents of tobacco subjected to different NH_4_^+^: K^+^ ratios. Graphs **(A–G)** represent increasing NH_4_^+^ at constant K^+^ (at 1) ratios. **(A)** NH_4_^+^/K^+^ ratio 1:1, **(B)** 2:1, **(C)** 5:1, **(D)** 10:1, **(E)** 20:1, **(F)** 50:1, and **(G)** 100:1. Graphs **(H–K)** connote constant NH_4_^+^ (at 1) at increasing K^+^ ratio. **(H)** NH_4_^+^/K^+^ ratio 1:2, **(I)** 1:5, **(J)** 1:10, **(K)** 1:20 (0.1-2 mM and 0.5-10 mM) and 1:50 (0.1-5 mM and 0.2-10 mM) **(L)** comparison of NH_4_^+^-K^+^ concentration within each ratio. All the NH_4_^+^/K^+^ concentration within each ratio were compared with the positive (without NH_4_^+^, but with K^+^). And negative control (without NH_4_^+^ and K^+^). Chlorophyll content was measured 15 days after treatment. Significant means were separated using standard deviation + SD (n= 12 biological replicates).

As shown in (**Figure 5**), there were significant differences in the chlorophyll content of tobacco subjected to concentration at different NH_4_^+^: K^+^ ratios. In each NH_4_^+^: K^+^ ratio, the chlorophyll content increased with increasing NH_4_^+^ - K^+^ concentration and was highest at 20-1 mM (20:1); a further increase in NH_4_^+^ ratio to 50:1 and 100:1 led to a gradual decrease in chlorophyll content. Compared with increasing NH_4_^+^/constant K^+^, constant NH_4_^+^/increasing K^+^- plants had lower chlorophyll content.

### Stem growth of tobacco seedlings as affected by varying NH_4_^+^/K^+^ concentration and ratios

Increasing NH_4_^+^ at constant K^+^ exerts varying effects on the fresh stem weight (**Figure 1**). Fresh stem weight increases with a progressive increase in NH_4_^+^-K^+^ concentration until a point was reached where further increase resulted in drastic stem reduction. Within this increasing NH_4_^+^/constant K^+^ ratio pattern, NH_4_^+^-K^+^ concentrations 2-2 mM (1:1) had the highest stem weight, followed by 2-1 mM (2:1) and 5-1 mM (5:1 mM), and their stem weight were increased by 56.4%, 13.2% and 7.1%, respectively, relative to the positive control. Positive control was considerably higher than all other treated concentrations at ratios 2:1 and 5:1, except at 2-1 mM (2:1) and 5-1 mM (5:1). Positive control had the highest stem weight at ratios 10:1, 20:1, 50:1, and 100:1. Indeed, stem weights of 50-1 mM (50:1) and 50-0.5 mM (100:1) were drastically reduced compared with the negative control. At constant NH_4_^+^/increasing K^+^, stem weight of all the concentrations at different ratios was remarkably higher than the positive control, though stem weight reduction was observed at 0.1-0.2 mM (1:2) and 0.1-0.5 mM (1:5). Compared with 2-2 mM (1:1; highest at constant K^+^ and increasing NH_4_^+^ ratio), the stem weight of 1-2 mM (1:2) and 1-5 mM (1:5) (highest stem weight under constant NH_4_^+^/increasing K^+^ ratio) increased by 21.4% and 17.4%, respectively.

Furthermore, there were significant differences in the stem K^+^ content at various ratios (**Figure 2**). At increasing NH_4_^+^ and constant K^+^, 2-2 mM (1:1) had the highest stem K^+^ content, followed by 10-5 mM (2:1), 5-1 mM (5:1) and 20-2 mM (10:1), exhibiting approximately 99.8%, 60.5%, 69.0% and 30.2%, increase in K^+^ content, respectively, relative to the positive control. However, compared with the positive control, stem K^+^ contents were significantly lower in concentration at ratios 20:1, 50:1, and 100:1. A 15.4% reduction in stem K+ content was observed in 50-0.5 mM (100:1) medium compared with negative control. Within each of the constant NH_4_^+^/increasing K^+^ ratios (except for ratio 1:20), increasing NH_4_^+^-K^+^ concentration led to gradual improvement in the stem K^+^ content until a peak was reached where further increase led to reduction. Generally, stem K^+^ content was highest at 1-5 mM (1:5), and was 1.2% higher than 2-2 mM (1:1).

Stem NH_4_^+^ contents were affected by various NH_4_^+^-K^+^ concentrations at different ratios (**Figure 3**). Under increasing NH_4_^+^/constant K^+^ ratios 1:1, 2:1, and 5:1, the NH_4_^+^ content of stem was much lower compared to 10:1, 20:1, 50:1, and 100:1 as visually represented in the graphs; however, a marked increase in stem NH_4_^+^content was observed when these treatments were compared with controls. Following this same increasing NH_4_^+^/K^+^ ratio pattern, stem NH_4_^+^ content peaks at the highest NH_4_^+^- K^+^ concentration. With the exemption of ratios 1:10 (0.2-2 mM), a similar increase in the stem NH_4_^+^ content was observed at highest NH_4_^+^-K^+^ concentration grown under constant NH_4_^+^/increasing K^+^ ratios. Interestingly, stem NH_4_^+^ content of such increasing K^+^ concentrations were drastically lower than most of the increasing NH_4_^+^/constant K^+^ treated medium (NH_4_^+^: K^+^ 20:1, 50:1, and 100:1).

**Figure 6:**
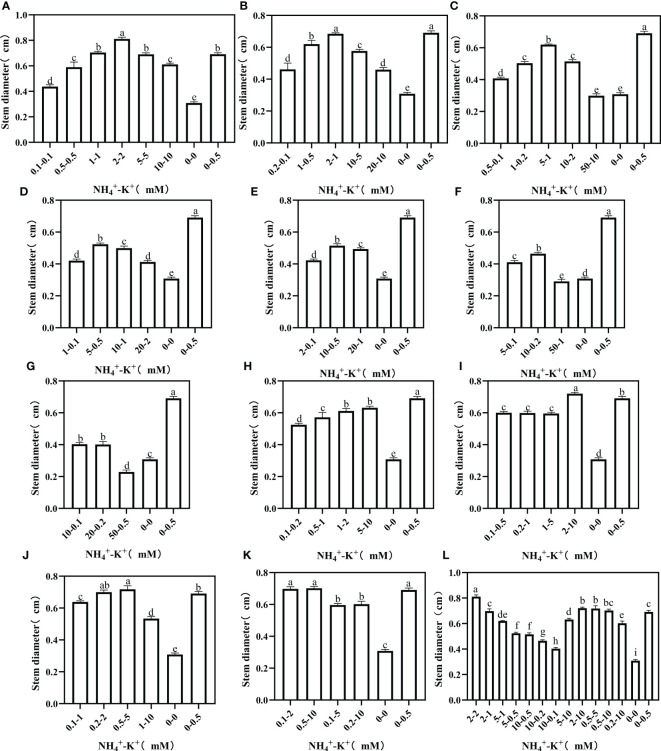
Stem diameter of tobacco as affected by different NH_4_^+^: K^+^ ratios. Graphs **(A-G)** represent increasing NH_4_^+^ at constant K^+^ (at 1) ratios. **(A)** NH_4_^+^/K^+^ ratio 1:1, **(B)** 2:1, **(C)** 5:1, **(D)** 10:1, **(E)** 20:1, **(F)** 50:1, and **(G)** 100:1. Graphs **(H–K)** connote constant NH_4_^+^ (at 1) at increasing K^+^ ratio. **(H)** NH_4_^+^/K^+^ ratio 1:2, **(I)** 1:5, **(J)** 1:10, **(K)** 1:20 (0.1-2 mM and 0.5-10 mM) and 1:50 (0.1-5 mM and 0.2-10 mM) **(L)** comparison of NH_4_^+^-K^+^ concentration within each ratio. All the NH_4_^+^/K^+^ concentration within each ratio were compared with the positive (without NH_4_^+^, but with K^+^).and negative control (without NH_4_^+^ and K^+^). The above growth parameters were measured 15 days after treatment. Significant means were separated using standard deviation ± SD (n= 3 biological replicates).

To further evaluate stem growth, stem diameter, which is one of the most important stem growth variables, was measured. At increasing NH_4_^+^/constant K^+^, stem diameter increases as the NH_4_^+^/K^+^ concentration increases; however, a point was reached where further increase led to a drop in stem diameter (**Figure 6**). There were significant reductions in the stem diameters of NH_4_^+^-K^+^ 50-10 mM (5:1), 50-1 mM (50:1), and 50-0.5 mM (100:1) compared with the negative control. In all the treated concentrations subjected to increasing NH_4_^+^ ratio, only 2-2 mM at ratio 1:1 had the largest stem diameter. Despite the increasing K^+^ at ratio 1:2, positive control still had the largest stem diameter. In case of further increase in K^+^ to 5 (1:5) and 10 (1:10), only 2-10 mM (1:5) and 0.5-5 mM (1:10) had larger stem diameter compared with positive control. The stem diameter of concentrations at ratios 1:20 and 1:50 was negligible since both had similar or smaller stem diameters compared with the positive control. In the overall treatments, 2-2 mM at ratio 1:1 had the largest stem diameter.

**Figure 7:**
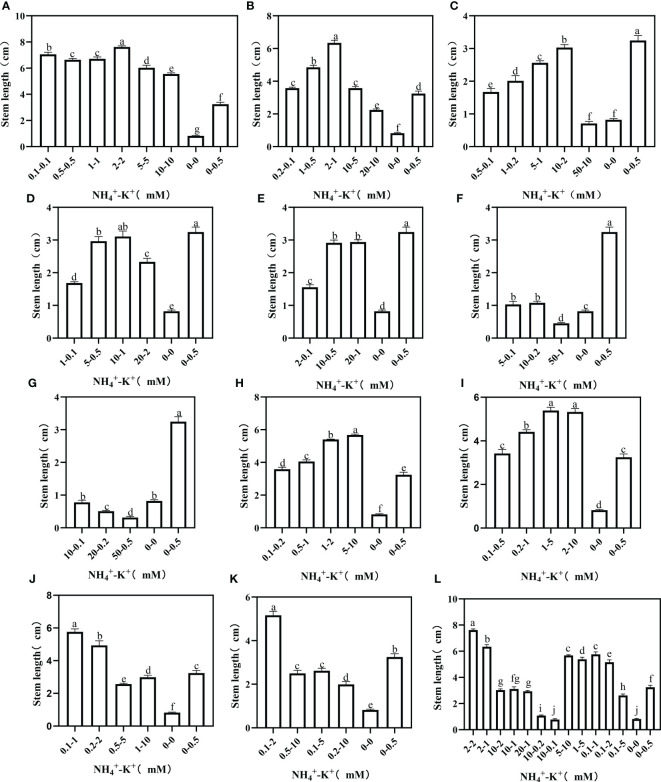
Stem length of tobacco as affected by different NH_4_^+^: K^+^ ratios. Graphs **(A-G)** represent increasing NH_4_^+^ at constant K^+^ (at 1) ratios. **(A)** NH_4_^+^/K^+^ ratio 1:1, **(B)** 2:1, **(C)** 5:1, **(D)** 10:1, **(E)** 20:1, **(F)** 50:1, and **(G)** 100:1. Graphs **(H–K)** connote constant NH_4_^+^ (at 1) at increasing K^+^ ratio. **(H)** NH_4_^+^/K^+^ ratio 1:2, **(I)** 1:5, **(J)** 1:10, **(K)** 1:20 (0.1-2 mM and 0.5-10 mM) and 1:50 (0.1-5 mM and 0.2-10 mM) **(L)** comparison of NH_4_^+^-K^+^ concentration within each ratio. All the NH_4_^+^/K^+^ concentration within each ratio were compared with the positive (without NH_4_^+^, but with K^+^).and negative control (without NH_4_^+^ and K^+^). The above growth parameters were measured 15 days after treatment. Significant means were separated using standard deviation ± SD (n= 3 biological replicates).

Stem length was measured to fully understand the optimal NH_4_^+^-K^+^ concentration required for stem growth. At increasing NH_4_^+^/constant K^+^, an increase in NH_4_^+^/K^+^ concentration led to a rapid surge in stem length, but further increase led to a decline (**Figure 7**). Compared with the positive control, stem length was markedly increased in all concentrations at ratios 1:1 and 2:1, except at concentration 20-10 mM (2:1), which had a 30.9% reduction. The stem length of the positive control was significantly higher than all other treatments under increasing NH_4_^+^ ratios (5:1, 10:1, 20:1, 50:1, and 100:1). Following the same ratio pattern, 50-1 mM and 20-0.2 mM (50:1) and 50-0.5 mM (100:1) were lower than the negative control. Regardless of both controls, stem length increases as NH_4_^+^/K^+^ concentration increases at ratios 1:2 and 1:5. Conversely, at ratios 1:10, 1:20, and 1:50, a drastic reduction in the stem length was observed when the concentration was increased. Taken together, stem length peaks at 2-2 mM (1:1), and was 35.1% higher than 5-10 mM (1:2) (which was the highest under constant NH_4_^+^/increasing K^+^ ratio).

### Root growth of tobacco seedlings as affected by varying NH_4_^+^/K^+^ concentration and ratios

Root weight differs within the treated concentrations at different ratios (**Figure 1**). Within each of the increasing NH_4_^+^: constant K^+^ ratios, fresh root weight increases with increasing NH_4_^+^ -increasing K^+^ concentration until a point was reached where further increase led to a gradual decrease. Compared with the positive control (1 mM K^+^ supply in the absence of NH_4_^+^), the fresh root weight of NH_4_^+^-K^+^ millimolar concentration 2-2 mM at ratios 1:1 was the highest, followed by 2-1 mM (2:1), with 37.1% and 8.2% increase, respectively. At ratios 5:1, 10:1, 20:1, 50:1, and 100:1, positive control had the highest root weight, but when compared with the negative control, which seems lower than all observed treatments, root weight of 50-0.5 mM at ratio 100:1 decreased by 36%. Except at lower NH_4_^+^ - K^+^ concentrations of constant NH_4_^+^/increasing K^+^ ratios, all other treated concentrations had increased root weight compared with the positive control. In all the concentrations examined, root weight peaks at 2-2 mM (1:1).

There were significant differences in the K^+^ content of roots under varying NH_4_^+^-K^+^ concentration, and are presented in **Figure 2**. A progressive increase in NH_4_^+^- K^+^ concentrations at increasing NH_4_^+^/constant K^+^ ratios resulted in a marked increase in the root K^+^ content, however, a point was reached where further increase led to a marked reduction. Except at lower NH_4_^+^-K^+^ concentrations (for example 0.1-0.1 mM, 0.2-0.1mM, 0.5-0.1mM, 1-0.2mM, 1-0.1mM and 2-0.1mM), the root K^+^ content of all other concentrations at NH_4_^+^: K^+^ ratios 1:1, 2:1, 5:1, 10:1, and 20:1 was significantly higher than the positive control. Root K^+^ content was highest at 10-5 mM (2:1), followed by 2-2 mM (1:1), 2-1 mM (2:1), 5-1 mM (5:1), 10-1 mM (10:1) and 20-1 mM (20:1). However, all concentrations at ratios 50:1 and 100:1 exhibit reduced K^+^ content relative to the positive control. Compared with the positive control, all observed concentrations under constant NH_4_^+^/increasing K^+^ ratios, had marked increment in their root K^+^ content, except 0.1-0.2 mM (1:2). At such NH_4_^+^/K^+^ ratio, root K^+^ content peaks at 1-5 mM (1:5), and increased by 26.4% relative to 10-5 mM (2:1), which was highest under increasing NH_4_^+^/constant K^+^ ratio.

There were significant differences in the NH_4_^+^ content of root under varying NH_4_^+^-K^+^ concentration, and are presented in **Figure 3**. At ratios 1:1, 2:1, 5:1, and 10:1 root NH_4_^+^ content increases with increasing NH_4_^+^- K^+^ millimolar concentration until a point was reached where a notable reduction in NH_4_^+^ content was observed (NH_4_^+^-K^+^ 2-2 mM (ratio 1:1); 2-1mM (2:1); 10-2mM (5:1); 10-1 mM (10:1); 10-0.2 mM (50:1)). A further increase in NH_4_^+^-K^+^ millimolar concentration resulted in a marked increase in leaf NH_4_^+^ content. However, at ratios 20:1 and 100:1, leaf NH_4_^+^ content increases with increasing NH_4_^+^-K^+^ concentration. Following same increasing NH_4_^+^ ratio pattern, positive control had the lowest root NH_4_^+^ content. Regardless of both controls, at constant NH_4_^+^/increasing K^+^ ratios, a direct relationship was observed between the root NH_4_^+^ content and NH_4_^+^/K^+^ concentration, except 1-20 mM (1:20). Under the same ratio pattern, treatment with no K^+^ and NH_4_^+^ (negative control) had the highest root NH_4_^+^ content.

**Figure 8:**
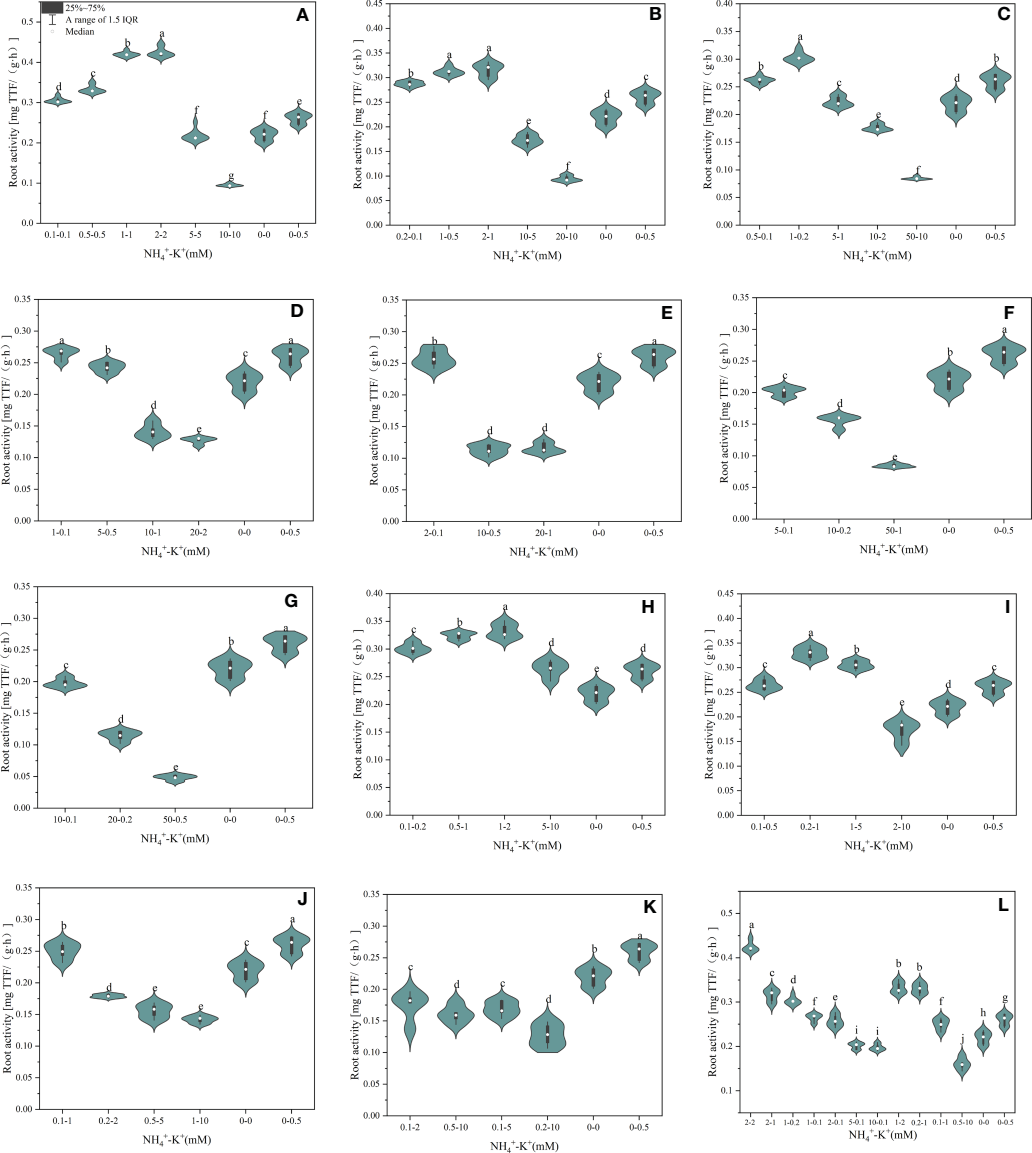
Root activity of tobacco as affected by different NH_4_^+^: K^+^ ratios. Graphs **(A–G)** represent increasing NH_4_^+^ at constant K^+^ (at 1) ratios. **(A)** NH_4_^+^/K^+^ ratio 1:1, **(B)** 2:1, **(C)** 5:1, **(D)** 10:1, **(E)** 20:1, **(F)** 50:1, and **(G)** 100:1. Graphs **(H–K)** connote constant NH_4_^+^ (at 1) at increasing K^+^ ratio. **(H)** NH_4_^+^/K^+^ ratio 1:2, **(I)** 1:5, **(J)** 1:10, **(K)** 1:20 (0.1-2 mM and 0.5-10 mM) and 1:50 (0.1-5 mM and 0.2-10 mM) **(L)** comparison of NH_4_^+^-K^+^ concentration within each ratio. All the NH_4_^+^/K^+^ concentration within each ratio were compared with the positive (without NH_4_^+^, but with K^+^).and negative control (without NH_4_^+^ and K^+^). The above growth parameters were measured 15 days after treatment. Significant means were separated using standard deviation ± SD (n= 9 biological replicates).

As shown in **Figure 8**, root activity was affected by NH_4_^+^/K^+^ concentration at different ratios. At ratios 1:1, 2:1, and 5:1, root activity was enhanced as NH_4_^+^/K^+^ concentrations increased; however, a point was reached where further increase resulted in a decrease in root activity. Conversely, at NH_4_^+^/K^+^ ratios 10:1, 20:1, 50:1 and 100:1, root activity decreases with increasing NH_4_^+^/K^+^ concentrations. Concentrations 2-2 mM and 1-1 mM at ratios 1:1 had the strongest root activity. However, relative to the positive control, the root activity of other increasing NH_4_^+^ ratios (10:1, 20:1, 50:1, and 100:1) was either insignificant or decreased. At constant NH_4_^+^/increasing K^+^ ratio, root activity was strongest at concentrations 1-2 mM (1:2) and 0.2-1 mM (1:5), whereas, at NH_4_^+^/K^+^ ratios 1:10, 1:20 and 1:50, positive control had the highest root activity. Of all the treated concentration, root activity peaks at concentrations NH_4_^+^ - K^+^ 2-2 mM (1:1).

**Figure 9:**
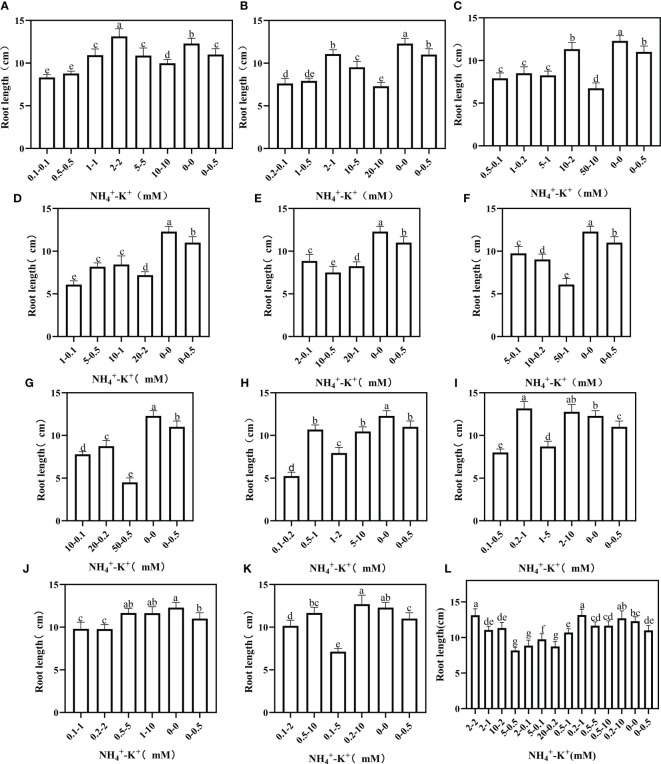
Root length of tobacco as affected by different NH_4_^+^: K^+^ ratios. Graphs **(A-G)** represent increasing NH_4_^+^ at constant K^+^ (at 1) ratios. **(A)** NH_4_^+^/K^+^ ratio 1:1, **(B)** 2:1, **(C)** 5:1, **(D)** 10:1, **(E)** 20:1, **(F)** 50:1, and **(G)** 100:1. Graphs **(H–K)** connote constant NH_4_^+^ (at 1) at increasing K^+^ ratio. **(H)** NH_4_^+^/K^+^ ratio 1:2, **(I)** 1:5, **(J)** 1:10, **(K)** 1:20 (0.1-2 mM and 0.5-10 mM) and 1:50 (0.1-5 mM and 0.2-10 mM) **(L)** comparison of NH_4_^+^-K^+^ concentration within each ratio. All the NH_4_^+^/K^+^ concentration within each ratio were compared with the positive (without NH_4_^+^, but with K^+^).and negative control (without NH_4_^+^ and K^+^). The above growth parameters were measured 15 days after treatment. Significant means were separated using standard deviation ± SD (n= 3 biological replicates).

The effects of variations in NH_4_^+^-K^+^ concentration at different ratios were further evaluated in root length. The root length of the negative control was significantly higher than all other treated concentrations at an increasing NH_4_^+^/constant K^+^ ratio; only the root length of 2-2 mM at ratio 1:1 increased by 6.8% relative to the negative control (**Figure 9**). Similarly, at constant NH_4_^+^/increasing K^+^ ratios, only concentration 0.2-1 mM (1:5) had markedly increased (13.16 cm) root length compared with the negative control (12.29 cm), all other observed concentrations had a pronounced reduction in root length.

**Figure 10:**
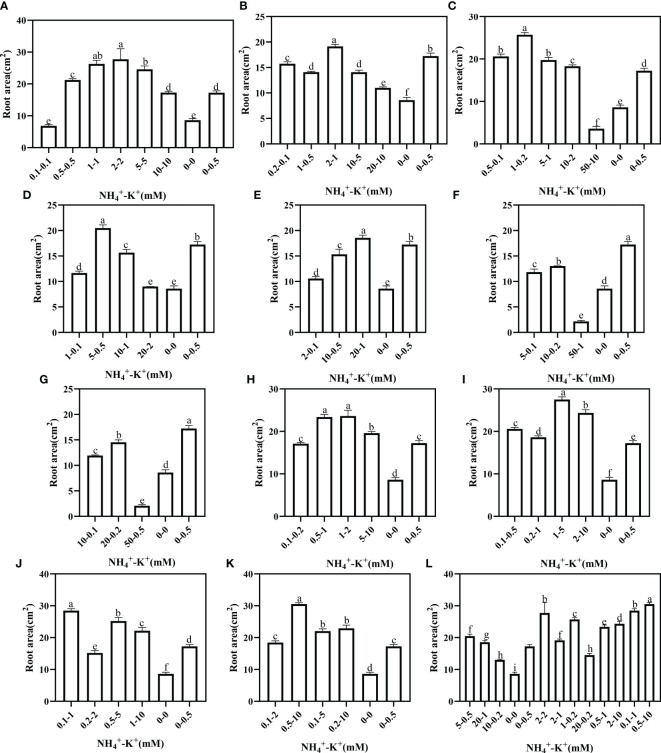
Root area of tobacco as affected by different NH_4_^+^: K^+^ ratios. Graphs **(A-G)** represent increasing NH_4_^+^ at constant K^+^ (at 1) ratios. **(A)** NH_4_^+^/K^+^ ratio 1:1, **(B)** 2:1, **(C)** 5:1, **(D)** 10:1, **(E)** 20:1, **(F)** 50:1, and **(G)** 100:1. Graphs **(H-K)** connote constant NH_4_^+^ (at 1) at increasing K^+^ ratio. **(H)** NH_4_^+^/K^+^ ratio 1:2, **(I)** 1:5, **(J)** 1:10, **(K)** 1:20 (0.1-2 mM and 0.5-10 mM) and 1:50 (0.1-5 mM and 0.2-10 mM) **(L)** comparison of NH_4_^+^-K^+^ concentration within each ratio. All the NH_4_^+^/K^+^ concentration within each ratio were compared with the positive (without NH_4_^+^, but with K^+^).and negative control (without NH_4_^+^ and K^+^). The above growth parameters were measured 15 days after treatment. Significant means were separated using standard deviation ± SD (n= 3 biological replicates).

Under increasing NH_4_^+^/constant K^+^, root area was highest at 2-2 mM (1:1), 2-1 mM (2:1), 1-0.2 mM (5:1), 5-0.5 mM (10:1) and 20-1 mM (20:1), but when compared with the positive control, all concentrations at ratios 50:1 and 100:1 had significantly lower root area (**Figure 10**). At constant NH_4_^+^/increasing K^+^ ratio, only 0.2-2 mM at ratio 1:10 had a lower root area relative to the positive control; all other observed concentrations under such increasing K^+^ ratio pattern was significantly higher. In all the treated concentrations, the root area peaks at 0.5-10 mM (1:20).

### Correlation among the growth parameters

**Figure 11:**
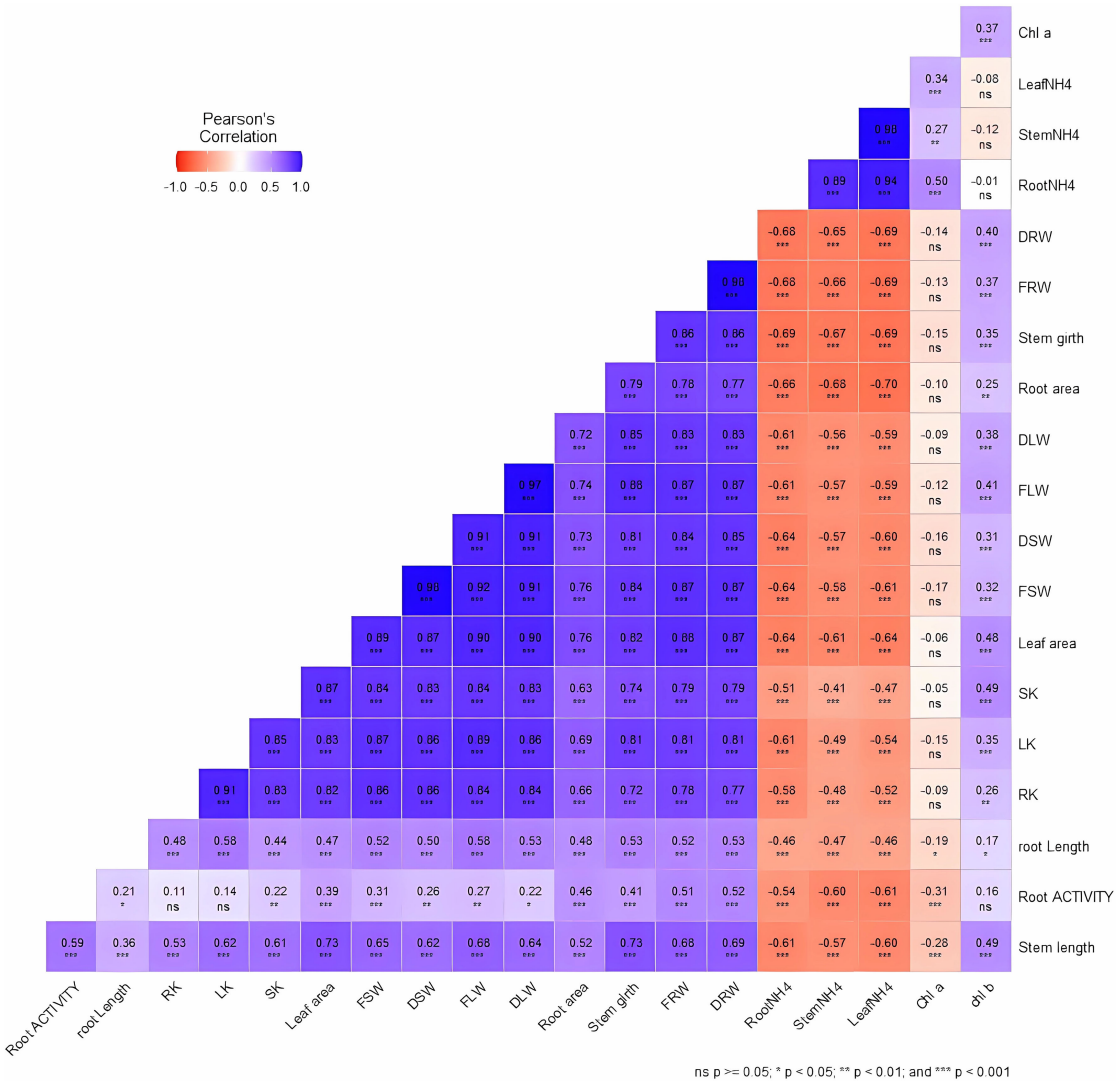
Relationship between growth variables in tobacco leaves, stems, and roots. The correlation analysis was performed using pearson correlation in R studio. ns: no significance difference; *p < 0.05; **p < 0.01; ***p < 0.001. The growth variables is represented by the acronyms in the figure, which include, dry root weight (DRW), fresh root weight (FRW), stem girth (diameter), root area, dry leaf weight (DLW), fresh leaf weight (FLW), dry stem weight (DSW), fresh stem weight (FSW), leaf area, stem K^+^ content (SK), leaf K^+^ content (LK), root K^+^ content (RK), chlorophyll a (Chl a), chlorophyll b (Chl b), leaf NH_4_^+^ content (LeafNH4), stem NH_4_^+^ content (stem NH4), root NH_4_^+^ content (root NH4), root length, root activity, and stem length.

We performed correlation analysis to establish the relationship between different growth parameters and their contributions to tobacco performance under different NH_4_^+^/K^+^ concentrations (**Figure 11**). Most of the growth traits are either positively or negatively correlated, very few are not significantly correlated. The strongest negative correlations (*p* < 0.001) are obtained between NH_4_^+^ content (leaves, stems and roots) and the other growth indicator such as dry root weight (DRW), fresh root weight (FRW), stem girth (diameter), root area, dry leaf weight (DLW), fresh leaf weight (FLW), dry stem weight (DSW), fresh stem weight (FSW), leaf area, stem K^+^ content (SK), leaf K^+^ content (LK), root K^+^ content (RK), root length, root activity, and stem length. This implies that value increase of NH_4_^+^ content in the leaves, stems, and roots leads to a decrease in each of the listed growth variables. However, nearly all other growth variables are positively correlated with each other. For example, increase in SK led to an increase in leaf area (*p* < 0.001), fresh weights (*p* < 0.001), among others. The relationship further supported the notion that excessive NH_4_^+^ supply leads to plant growth retardation.

## Discussion

The influence of K^+^ and nitrogen forms (NH_4_^+^ and NO_3_^-^) on plant growth and development has been a focus of research (Wang et al., 2003; Szczerba et al., 2008; Zhang et al., 2010; Witold et al., 2017). However, no such research has addressed the appropriate quantity of K^+^ and NH_4_^+^ required to enhance plant growth. To fully understand the effects of varying NH_4_^+^/K^+^ concentration on growth, and the appropriate amount required for optimal growth of tobacco seedlings, the 15-day- NH_4_^+^/K^+^ treated samples were evaluated on a leaf, stem and root basis. Our study revealed that NH_4_^+^- K^+^ concentration had a profound effect on the growth of tobacco seedlings while pinpointing the right combination needed for the optimum seedling growth.

### Leaf parameter

It has been shown that potassium (K^+^) and nitrogen (N) (ammonium (NH_4_^+^) as a major form of inorganic nitrogen) is the most limiting nutrient during plant growth (Szczerba et al., 2008; El Gendy et al., 2015). Thus, symptoms ensued from their excessive supply/deficiency are notable on fresh leaf weight (**Figure 1**), dry leaf weight (**Figure S1**) leaf NH_4_^+^ and K^+^ content (**Figures 2**, **3**), leaf area (**Figure 4**), leaf color (**Figure S2**) and chlorophyll content (**Figure 5**). The supply of NH_4_^+^ and K^+^ concentrations to tobacco plants during the seedling stage exerted varying effects on leaf weight. Under increasing NH_4_^+^ at constant K^+^ ratios, the tolerance of tobacco leaf to increasing NH_4_^+^/K^+^ nutrition was optimal at ratios 1:1 (2-2mM, which had the highest fresh leaf weight; 11.2 g/plant), 2:1(2-1 mM; 8.6 g/plant) and 5:1 (5-1 mM; 7.6 g/plant), and beyond these ratios, the tolerance capacity decreased drastically (**Figure 1**). However, the study by Balkos et al. (2010) revealed optimum leaf growth in rice plants with NH_4_^+^- K^+^ concentration 10-5 mM (ratio 2:1), suggesting strong tolerance of rice to high NH_4_^+^ nutrition, indicating that plant tolerance to high NH_4_^+^ is plant species dependent. Here, NH_4_^+^: K^+^ ratios beyond 5:1 impaired leaf growth, leading to stunted leaf weight and shrank leaves with deep greenish or yellowish colouration (leaf chlorosis). These symptoms, as presented in **Figure S2**, are typical of NH_4_^+^ toxicity. Also, at NH_4_^+^: K^+^ ratios 10:1, 20:1, 50:1 and 100:1, NH_4_^+^ toxicity repressed leaf growth, and reduced leaf K^+^ content (**Figure 2**). Following the same ratio pattern (increasing NH_4_^+^/constant K^+^), for example, NH_4_^+^/K^+^ millimolar concentration 2-2 mM at ratio 1:1 had improved leaf weight (11.2 g/plant) and K^+^ content (1.76 mmolg/DW), whereas, a drastic leaf weight (3.37 g) and K^+^ content (0.49 mmolg/DW) reduction was observed in NH_4_^+^: K^+^ ratio 20:1 (2-0.1 mM) (**Figures 1**, **2**). This result indicates that leaf growth and K^+^ content are optimum at equal NH_4_^+^/K^+^ ratios 1:1, but are suppressed beyond ratios 5:1 during the seedling stage (**Figures 1**, **2**). This is supported by previous studies, which reported that excessive supply of NH_4_^+^ nutrient represses leaf growth and K^+^ uptake (Szczerba et al., 2006; Shi et al., 2020a; Aluko et al., 2022). It is worth noting that the reduced K^+^ content in the leaf could be linked to NH_4_^+^ toxicity. Samples with high NH_4_^+^ tends to have higher leaf NH_4_^+^ content and lower leaf K+ content (**Figure 11**), an indication that an appropriate amount of these two nutrients is required for growth. Perhaps, the strong negative correlation between NH_4_^+^ toxicity and reduced K^+^ content in plant tissue altered in the balance between these two cations (NH_4_^+^ and K^+^) (Shi et al., 2020a).

Interestingly, beyond the optimal NH_4_^+^ - K^+^ concentrations (at increasing concentrations of both nutrients (NH_4_^+^ and K^+^), leaf weight decreases. Using ratio 1:1 as a good example, tobacco leaf weight at NH_4_^+^- K^+^ concentrations 5-5 mM and 10-10 mM, decreases by 37.5% and 43.5%, respectively, relative to the NH_4_^+^- K^+^ 2-2 mM (where optimal leaf weight was attained) (**Figure 1**). It appears that excessive supply of NH_4_^+^ and K^+^ beyond the optimal triggers leaf weight reduction, and in line with Shi et al., 2020a, who reported the same for *Arabidopsis*. The reductions in leaf growth under such high K^+^ and NH_4_^+^ conditions may partly be due to the energetic drain on root cells catalyzing substantial futile cycling of both cations (K^+^ and NH_4_^+^) when nutrient supply is high (Britto and Kronzucker, 2001; Britto and Kronzucker, 2002; Szczerba et al., 2006). Also, low concentrations of NH_4_^+^ and K^+^ may adversely affect leaf growth. In the present study, low NH_4_^+^- K^+^ concentrations (0.1-0.1 mM, 0.1-0.2 mM) had significantly reduced fresh leaf weight (**Figures 1**, **11**). This result demonstrated that K^+^ and NH_4_^+^ deficiency also impair leaf growth (Shi et al., 2020a; Li et al., 2021; Liu et al., 2022), and as such, maintaining an optimal nutritional balance between these two cations becomes expedient.

NH_4_^+^ toxicity was mitigated with an additional K^+^ supply to wheat plants (Guo et al., 2019). Similarly, Kong et al. (2014) revealed that an extra supply of K^+^ mitigated the detrimental effects of excessive supply of NH_4_^+^, thus increasing the culm mechanical strength and N remobilization efficiency of wheat by 23% and 35%, respectively. Our findings showed that maintaining NH_4_^+^ and K^+^ at appropriate concentrations rather than an excessive supply of NH_4_^+^, could reduce the incidence of stunted leaf weight and leaf chlorosis. Given that leaf weight and leaf K^+^ content peaks at NH_4_^+^- K^+^ concentration 2-2 mM (at ratio 1:1), increased leaf weight at this concentration is strongly associated with the leaf K^+^ content (**Figure 11**) (Santos et al., 2021). However, at increasing K^+^ concentrations, the highest leaf weight (11.5 g) was attained at 5-10 mM and was comparable to that observed under 2-2mM (11.2 g) NH_4_^+^ - K^+^ concentration (**Figure 1L**), demonstrating the putative role of K^+^ in leaf development. In addition, NH_4_^+^: K^+^ at equal ratios (1:1) enhances the fresh leaf weight of tobacco seedlings. A similar trend was reported in *Arabidopsis*, with optimal shoot weight attained at NH_4_^+^: K^+^ ratio 1:1 (0.5-0.5 mM) (Shi et al., 2020a).

Leaf surface area is a crucial parameter that determines the capacity of a crop to intercept photosynthetic light, thus, affecting leaf growth and productivity (Weraduwage et al., 2015). Reduction in leaf area was more severe under a high NH_4_^+^: constant K^+^ ratio (**Figure 4**), thus limiting leaf growth under such conditions. Similar findings have been reported in tobacco (Weraduwage et al., 2015), wheat (Guo et al., 2019), and sugar beet (Raab and Terry, 1994). This finding showed that there is a strong correlation between reduced leaf area and leaf growth; hence the inhibition of tobacco leaf growth could be attributed to a reduction in leaf area (**Figure 11**). However, at increasing K^+^ concentration, increased leaf area suggests the crucial role of K^+^ in leaf expansion (**Figures 4**, **11**) (Hu et al., 2020).

Based on the visual evaluation, leaf chlorophyll content is often assessed in terms of leaf colour. In the present study, we observed that the leaf colour of high NH_4_^+^-fed seedlings was dark green (**Figure S2**), and consequently, higher chlorophyll content was observed in such plants (**Figures 5** , **11**). This is in line with the study of Bojović and Marković (2009), which revealed that plants exposed to high N have leaves with dark green colour. The dark green leaf colour and enhanced chlorophyll content are associated with N being a structural component of chlorophyll, thereby influencing chloroplast formation in plants. Thus, leaf colour is strongly associated with the N content of the leaf. Nonetheless, under high NH_4_^+^ and constant K^+^ ratios 50:1 and 100:1, a drastic decline in the chlorophyll content was observed, reflecting leaf chlorosis (**Figures S2**, **5**). Light green colouration was observed in the leaves of constant NH_4_^+/^increasing K^+^ - fed plants (**Figure S2**; Bojović and Marković (2009) reported that cultivars with low N content had reduced chlorophyll content in their leaves. Hence, reductions in the intensity of leaf green colour could be due to the reduced N content and, consequently low chlorophyll content.

We deduced that optimal growth for tobacco leaf during the seedling stage could be achieved with NH_4_^+^ and K^+^ at concentrations of 2-2 mM (ratio 1:1) (**Figure 1L**). This is because at such concentration, all leaf growth variables were significantly improved compared to positive control. Thus, any further increase or decrease beyond such concentration might not be crucial for tobacco leaf growth at the seedling stage. From our findings, the severity of K^+^ and NH_4_^+^ symptoms due to excessive supply or deficiency was apparent on the leaf growth parameters, which is in line with previous studies (Shi et al., 2020a; Li et al., 2021; Poucet et al., 2021; Saloner and Bernstein, 2022).

### Stem parameter

Stem growth often depends on the availability of nutrients such as N and K^+^ (Souza and Tavares, 2021; Xie et al., 2021; Aluko et al., 2022; Marschner and Rengel, 2023). Therefore, stem growth must be ensured to facilitate photosynthates and uptake of nutrient through the root, hence, the need for optimal supply of NH_4_^+^ - K^+^ at the right concentration and ratio.

Various NH_4_^+^ - K^+^ concentrations at different ratios influence stem growth. Our findings showed that thin stems were evident under high NH_4_^+^/constant K^+^, especially at ratios beyond 5:1 (**Figures 1**, **S2**), which suggests that the stem biomass was significantly suppressed due to NH_4_^+^ toxicity. In addition, stem weight was optimal at 1-2 mM (1:2), demonstrating the preference of stem for K^+^ over NH_4_^+^. This result indicates that tobacco stems are more susceptible to an excessive supply of NH_4_^+^, unlike the leaf weight, which was optimal at 2-2 mM (**Figure 1**).

Moreover, stem K^+^ content and stem growth were reduced when plants were exposed to NH_4_^+^/K^+^ ratios above 10:1, while NH_4_^+^ stem content was increased (**Figures 2**, **11**). This observation was not unexpected as an excessive supply of NH_4_^+^ inhibits K^+^ uptake. Our research findings are in accordance with earlier studies in rice (Szczerba et al., 2008), barley (Walch-Liu et al., 2000), and wheat (Guo et al., 2019), that high NH_4_^+^ nutrition reduces K^+^ uptake and content. The effects of NH_4_^+^ toxicity was obviated with the supply of K^+^ at an increased concentration over NH_4_^+^. However, at high NH_4_^+^ - K^+^ concentrations [e.g., 20-2 mM and 10-1 mM (at ratio 10:1)] of both nutrients, K^+^ content was higher than the positive control (without NH_4_^+^) (**Figure 2**), probably because the K^+^ concentration was sufficient to counteract the effect of excessive NH_4_^+^. Furthermore, our results showed that at concentrations beyond 5-1 mM (at ratio 5:1), a decrease in stem growth was observed, but the K^+^ content was still high at ratio 10:1 (e.g. 20-2 mM and 10-1 mM) (**Figures 1**, **2**), which indicates that tobacco stem can tolerate excess K^+^ up to ratios 10:1. The reason for such findings could partly be that under high nutrient supply, there is diversion of energy needed for growth to recycle excessive K^+^ and NH_4_^+^ which culminates in poor nutrient utilization due to energy drain and negative feedback mechanism (Britto et al., 2001; Britto and Kronzucker, 2002; Britto and Kronzucker, 2006). Unexpectedly, at constant NH_4_^+^/increasing K^+^ concentration, a drop in K^+^ content was notable at; 1-2 mM, 1-5 mM and 0.5-5 mM (**Figure 1**). A similar reduction was observed when external K^+^ supply was raised from 1.5 mM to 40 mM in rice seedlings (Szczerba et al., 2008). This interesting phenomenon may suggest the crucial need for a nutritional balance between these ions (NH_4_^+^ and K^+^) for plant growth and development.

We investigated the effect of NH_4_^+^ and K^+^ treatments on stem length and diameters, which are crucial indicators for stem growth. Our results showed that stem diameter was suppressed at ratios exceeding NH_4_^+^: K^+^ ratios 1:1 (**Figure 6**), while stem length was reduced at NH_4_^+^: K^+^ ratios beyond 2:1 (**Figure 7**). Also, stem length and stem diameter were increased at an elevated K^+^ concentration of 5 (NH_4_^+^: K^+^ ratio 1:5) but decreased when K^+^ exceeded such limit. These findings further demonstrated that stem growth could be enhanced when there is a balance between these ions (NH_4_^+^: K^+^). Zaman et al. (2015) mitigated the negative impact of excessive N application by supplementing with external K^+^ to improve stem strength and yield. Following the obtained result from stem weight and other growth variables, stem growth appears optimal at NH_4_^+^ - K^+^ concentrations 1-2 mM or 2-2 mM.

### Root parameter

The root is the major plant organ responsible for the uptake of water and other mineral nutrients, including K^+^ and NH_4_^+^; hence, it becomes imperative to understand the optimal NH_4_^+^ - K^+^ concentrations required for the proper functioning of the root. The present study evaluated the root growth of tobacco plants at the seedling stage based on their exposure to NH_4_^+^ and K^+^ nutrients at various concentrations. Reductions in root growth evident in thin root and reduced K^+^ content was observed in increasing NH_4_^+^ - constant K^+^ concentrations beyond 5-1 mM (5:1), suggesting visible symptoms of NH_4_^+^ toxicity (**Figures 1**, **S2**). In support of this claim, Banuelos et al. (2002) reported reduced K^+^ uptake in detached rice roots exposed to high NH_4_^+^ concentration. The negative effect observed with high NH_4_^+^ nutrition was obviated when external K^+^ was increased (**Figure S2**), suggesting that NH_4_^+^ supply in plants depend on the optimal supply of K^+^ (Shi et al., 2020a).

Also, root growth variables, including root activity, root length, and root area, were improved with increasing K^+^ ratios (**Figures 8**-**10**). Although increasing K^+^ exerts a positive effect on root growth, optimal root growth was attained at equal concentrations of NH_4_^+^- K^+^ 2-2 mM (1:1). The response of various parts of tobacco plants organs (leaf, stem, and root) are influenced by both NH_4_^+^ **-** K^+^ millimolar concentration and ratio at which the nutrient was supplied. This is the first study that examined such influence, regarding NH_4_^+^: K^+^ ratios and concentration to ascertain optimum growth. This justifies why nutrient supply cannot be based only on either concentration or ratio, but on a combination of both provides an in-depth understanding of the optimal growth of tobacco seedlings.

## Conclusion

Our study showed that various parts of the tobacco plant (leaf, stem, and root) respond differently to varying concentrations of NH_4_^+^/K^+^ as well as ratios. Optimum growth of tobacco leaf and root were observed with equal concentrations of NH_4_^+^/K^+^ (2-2 mM) at ratio 1:1, whereas stem growth was attained with concentration of 1 mM NH_4_^+^ and 2 mM K^+^ at ratio (1:2). Interestingly, our results explained the degree of tolerance of different organs of tobacco plant to ammonium despite its natural low tolerance for ammonium. This novelty was not explained in previous research on other crops which have low tolerance for ammonium like tobacco, rather the investigations was concentrated on the physiological response of NH_4_^+^ to plant yield. The study provided an insight into the right combination of NH_4_^+^/K^+^ that could mitigate or prevent NH_4_^+^ or K^+^ stress in the tobacco seedlings. In the same lieu, the large sample size (47 treatments) at 12 NH_4_^+^/K^+^ ratios lends an evidence for determination of optimal concentration of NH_4_^+^/K^+^ required for growth of tobacco seedling in a hydroponic system. Although, this present study found that NH_4_^+^/K^+^ concentrations stated above would be required for optimal growth of different organs of tobacco plant at seedling stage, further research would be required to validate the optimal NH_4_^+^ - K^+^ concentration, and at what ratio, required for growth at a later developmental stage of tobacco plant.

## Data availability statement

The original contributions presented in the study are included in the article **Supplementary Material**. Further inquiries can be directed to the corresponding authors.

## Author contributions

CL and OA, designed the experiment, carried out most experiments, data analysis, and wrote the manuscript. SS participated in the design and provided useful advice. ZM, TN, CS, and ZL participated in the sample collection and parameter measurements. QW and HL conceived of the study and participated in its design and coordination. All authors contributed to the article and approved the submitted version.

## Supplementary material

Supplementary Figure 1 Dry weight of leaf (FLW), stem (FSW), and root (FRW) of tobacco plants as affected by different NH_4_^+^/K^+^ ratios. Graphs **(A-G)** represent increasing NH_4_^+^ at constant K^+^ (at 1) ratios. **(A)** NH_4_^+^/K^+^ ratio 1:1, **(B)** 2:1, **(C)** 5:1, **(D)** 10:1, **(E)** 20:1, **(F)** 50:1, and **(G)** 100:1. Graphs **(H-K)** connote constant NH_4_^+^ (at 1) at increasing K^+^ ratio. **(H)** NH_4_^+^/K^+^ ratio 1:2, **(I)** 1:5, **(J)** 1:10, **(K)** 1:20 (0.1-2 mM and 0.5-10 mM) and 1:50 (0.1-5 mM and 0.2-10 mM). **(L)** comparison of NH_4_^+^-K^+^ concentration within each ratio. All the NH_4_^+^/K^+^ concentration within each ratio were compared with the positive (without NH_4_^+^, but with K^+^).and negative control (without NH_4_^+^ and K^+^). Dry weight was determined 15 days after treatment. Letters represent the mean values ± SD (n= 15 biological replicates). The bars without letters have extremely low mean values.

Supplementary Figure 2 Tobacco seedlings subjected to various NH_4_^+/^K^+^ concentrations at different ratios. Plants were grown in a hydroponic system with all nutrients, including 1.4 mM NO_3_^-^ as N source but without NH_4_^+^ or K^+^ for 6 days and subsequently treated with different NH_4_^+^ and K^+^ for 15 days as indicated.
