# Supplementary figures and images for: Determination of optimal NH4 +/K + concentration and corresponding ratio critical for growth of tobacco seedlings in a hydroponic system
Source: Front Plant Sci. 2023 Jul 11;14:1152817. doi: 10.3389/fpls.2023.1152817 (PMC10368480; doi:10.3389/fpls.2023.1152817)

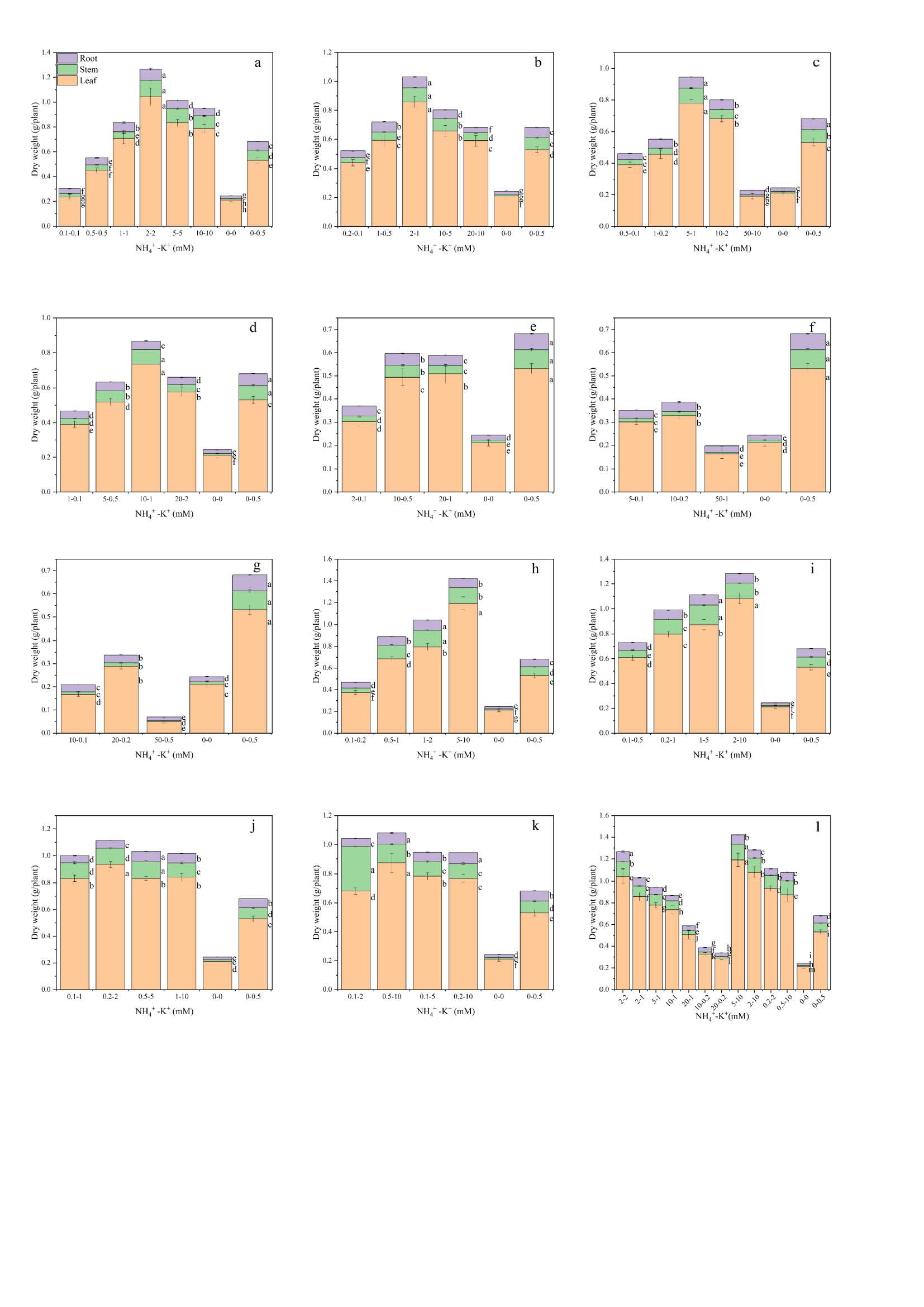

Supplement: Supplementary Figure 1 — Dry weight of leaf (FLW), stem (FSW), and root (FRW) of tobacco plants as affected by different NH4 +/K+ ratios. Graphs (A-G) represent increasing NH4 + at constant K+ (at 1) ratios. (A) NH4 +/K+ ratio 1:1, (B) 2:1, (C) 5:1, (D) 10:1, (E) 20:1, (F) 50:1, and (G) 100:1. Graphs (H-K) connote constant NH4 + (at 1) at increasing K+ ratio. (H) NH4 +/K+ ratio 1:2, (I) 1:5, (J) 1:10, (K) 1:20 (0.1-2 mM and 0.5-10 mM) and 1:50 (0.1-5 mM and 0.2-10 mM). (L) comparison of NH4 +-K+ concentration within each ratio. All the NH4 +/K+ concentration within each ratio were compared with the positive (without NH4 +, but with K+).and negative control (without NH4 + and K+). Dry weight was determined 15 days after treatment. Letters represent the mean values ± SD (n= 15 biological replicates). The bars without letters have extremely low mean values. [file DataSheet_1.zip › Figure S1 .JPEG]
